# Examining Alterations in Electroencephalograms in a Sample of Egyptian Children Suffering with ADHD

**DOI:** 10.1192/j.eurpsy.2025.1143

**Published:** 2025-08-26

**Authors:** M. M. Hamouda

**Affiliations:** 1 psychiatry, Al-Azhar faculty of medicine, Cairo, Egypt

## Abstract

**Introduction:**

A frequent developmental problem known as attention deficit hyperactivity disorder causes inattention, which may or may not be accompanied by hyperactivity.

Having trouble focusing, engaging in excessive activity, and acting in ways that are inappropriate for one’s age are all signs of ADHD.In the United States, research on the use of EEG to diagnose ADHD is still underway. The FDA has authorized the use of EEG to assess the illness’s morbidity alone.According to a different study, the EEG theta/beta ratio cannot definitively distinguish between people who have ADHD and those who do not.

**Objectives:**

the purpose of this study was to determine whether children with ADHD had altered EEGs.

**Methods:**

study was carried out from January to June 2024 on sixty non-epileptic children with ADHD (8–18 years old) at Al Hussian university hospital, Egypt.

This study was authorized by the Al-Azhar Faculty of Medicine’s Ethical Committee. Following an explanation of the purpose of the study and the acquisition of verbal agreement, all children underwent semi-structured clinical interviews and were excluded from any other neuropsychiatric or medical disorders. All study children have been diagnosed with ADHD according to the DSM IV criteria SCID, Conner’s scale for ADHD was applied for all the study’s children. EEG was obtained for all the children in the study. SPSS version 20.0 was employed. Numbers and percentages were used to characterize the qualitative data, and the significance of the outcomes was assessed at the 5% level.

**Results:**

The research involved 60 children with ADHD diagnoses, of whom 70% were younger than 8 years old, 71.7% of whom were male, 28.3% of whom were female, and 53.3% of whom were in rural settings.Based on study statistics, the mixed form of ADHD was the preponderance kind, with 56.7% of participants having ADHD. Oppositional defiant disorder (ODD) was a co-morbid disorder. the study indicates a strong statistical correlation (P value<0.001) between the combined type of ADHD and the individuals’ educational attainment.Accompanied conditions and male ADHD children were shown to have a high statistical significance (P<0.005), as was age less than 8 years old (P=0.026)

Only 10% of the non-epileptic ADHD children in the study had aberrant EEG readings, and there was no statistical correlation between them. there is a significant correlation (P<0.007) between co-morbid conditions and ADHD children, irrespective of their kind.

**Table:** 

	Hyperactivity and Attention deficit						c2	MCp
	Hyperactivity (n = 18)		Low Attention (n= 8)					
	No.	%			No.			
**EEG**							2.357	0.307
Changes	1	5.6	2	25.0	3	8.8		
Normal	17	94.4	6	75.0	31	91.2		
**Co morbid disorder**							13.003*	0.007*
No	5	27.8	0	0.0	5	14.7		
Conducted	10	55.6	3	37.5	7	20.6		
ODD	3	16.7	5	62.5	22	64.7

**Attention deficit Hyperactivity, EEG and accompanied disorder**

**Conclusions:**

The diagnosis of ADHD in children was not strongly correlated with changes in EEG.

**Disclosure of Interest:**

None Declared

